# Approaches of stem cell mobilization in a large cohort of metastatic germ cell cancer patients

**DOI:** 10.1038/s41409-022-01614-9

**Published:** 2022-02-21

**Authors:** Ramin Madanchi, Nils W. Engel, Winfried Alsdorf, Christoph Oing, Christian Frenzel, Finn-Ole Paulsen, Carsten Bokemeyer, Christoph Seidel

**Affiliations:** 1 Department of Oncology, Hematology and Bone Marrow Transplantation with Division of Pneumology, University Medical Center Hamburg-Eppendorf, Hamburg, Germany; 2 Mildred Scheel Career Center HaTriCS4, University Cancer Center Hamburg, University Medical Center Hamburg-Eppendorf, Hamburg, Germany

**Keywords:** Chemotherapy, Stem-cell therapies

## Abstract

High-dose chemotherapy (HD-Cx) in refractory germ cell cancer (GCC) is effective but limited data are available concerning the optimal approach for stem cell mobilization (SCM) in these patients. In this analysis 102 patients undergoing SCM during first (*n* = 25) or subsequent treatment lines (*n* = 77) were analyzed. Subcutaneous injections of granulocyte colony-stimulating factor (G-CSF) were given once daily (group 1) in 52 patients (51%), twice daily (group 2) in 39 patients (38%) or one injection Pegylated-G-CSF (PegG-CSF) (group 3) in eleven patients (11%) after one cycle of mobilization chemotherapy. Plerixafor was administered 13 times in group 1, seven times in group 2 and once in group 3. Overall, 77 (75%) patients achieved successful SCM defined as ≥8*10^6^ CD34+ cells/kg body weight for three consecutive HD-Cx plus one backup dose. In group 1, 40 of 52 patients (77%) achieved successful SCM with a median of 11 G-CSF injections, in group 2, 27 of 39 patients (69%) with a median of 14 G-CSF injections and in group 3, 10 of 11 patients (91%) with one injection of PegG-CSF. SCM was more successful if conducted during first-line chemotherapy (*p* = 0.016) and associated with a beneficial outcome concerning overall survival (*p* = 0.02) if performed satisfactorily.

## Introduction

Germ cell cancer (GCC) is the most common tumor type in young adults under the age of 40 with an increasing incidence over the past years [1–3]. Due to an excellent sensitivity to cisplatin-based chemotherapy and multimodal treatment approaches, cure rates of more than 80% can be achieved even in metastatic disease [4]. However, approximately 30% of primary metastatic patients relapse or progress despite platinum-based first-line treatment [5]. In this situation, the tumor cells can still be sensitive towards cytotoxic agents and salvage treatment with high-dose (HD) chemotherapy (Cx) followed by subsequent autologous stem-cell re-infusion has a curative potential associated with long-term survival rates of ~60% [6]. However, as refractory patients have received several treatment cycles prior to HD-Cx, mobilization failure rates of hematopoetic stem cells (HSCs) are predicted to be as high as 30%, correlating with the cumulative dose of cisplatinum applied during previous therapies [7]. Therefore, a standardized approach of stem cell mobilization (SCM) and apheresis is mandatory to generate the highest count and best quality of CD34 positive HSCs. For SCM, the administration of one to two cycles of induction chemotherapy followed by repetitive administrations of human granulocyte colony-stimulating factor (GCS-F) is considered as the standard of care [8, 9]. Alternatively, pegfilgrastim, a PEGylated long-lasting form of the recombinant human G-CSF with a half-life of 46–62 h can be administered as single injection [9]. In case of an insufficient amount of CD34 positive cells in the blood stream despite prior G-CSF stimulation, the additional administration of plerixafor, a C-X-C chemokine receptor type 4 (CXCR4) antagonist, can enhance the release of HSCs from the bone marrow to the peripheral blood stream [10, 11]. However, in Europe the use of plerixafor in combination with G-CSF is currently approved for lymphoma and multiple myeloma patients only, and data concerning efficacy in GCC patients are scarce. Moreover, multiple G-CSF administrations in combination with plerixafor generate high costs for the health care system. In this study, we reviewed the SCM approaches in a large cohort of GCC patients treated at a single GCC expert center looking for individual patient characteristics to be associated with the outcome concerning SCM. The prognostic impact and potential costs of successful SCM and patient characteristics were explored as well.

## Patients and methods

### Study population and inclusion criteria

We analyzed a consecutive cohort of 102 GCC patients receiving systemic chemotherapy and SCM at a high-volume center from the years 2008–2021. Monocentric data acquisition was in line with local requirements according to Hamburg Hospital Act (HmbKHG) § 12 HmbKHG and the declaration of Helsinki. All patients were included if they had (i) confirmed GCC histology according to histological examination by local pathologists, (ii) advanced disease including the clinical stages IIA-C and IIIA-C according to Union for International Cancer Control (UICC) [12] at the time of SCM, and (iii) SCM after systemic treatment with consecutive stimulation with G-CSF or Pegylated-G-CSF (PegG-CSF) administered subcutaneously.

### Outcome measurements and statistical analysis

The overall outcome measurement was successful SCM. According to our institutional standards HD-Cx was planned with three consecutive cycles of HD-Cx with cisplatin, ifosfamide and etoposide (HD-VIP) for primary high-dose chemotherapy or three cycles of carboplatin and etoposide (HD-CE) as salvage treatment in case of recurrence. With an additional stem cell fraction as backup dose the definition of successful SCM was to achieve four fractions of ≥2*10^6^ and a total rate of ≥8*10^6^ CD34+ cells/kg of body weight. Patients undergoing chemotherapy with consecutive SCM during 1st, 2nd or subsequent treatment lines were analyzed retrospectively regarding their SCM outcome and prognosis concerning overall survival (OS). OS was defined as the timepoint from first diagnosis until to the date of death or to the last day of follow-up. Potential correlations between successful SCM and (i) treatment line, (ii) type of growth factor used for CD34+ stem cell stimulation and (iii) patient baseline characteristics were investigated. To explore potential correlations between SCM outcomes with patient characteristics and mobilization treatment strategies, statistical analysis was conducted using Pearson Chi-square test. To explore patient characteristics concerning their prognostic impact regarding OS, survival analysis was conducted using the Kaplan–Meier method [13] and log-rank test was applied to compare survival rates. Variables were found to be significant if a two-sided *p* value was *<*0.05. If several patient characteristics were found to be associated with outcome concerning OS (*p* < 0.1) they would be tested in a multivariate Cox regression model.

## Results

### Patient characteristics

The median age was 33 years (range: 18–57) at primary diagnosis. At the time of mobilization chemotherapy with consecutive SCM disseminated disease was present in all patients. According to the IGCCCG stratification at the timepoint of metastatic disease nine patients (10%) were considered as good, 26 (25%) as intermediate and 67 (66%) as poor prognoses, respectively [14]. Further patient characteristics are given in Table 1.

**Table 1:** Patient characteristics.

Characteristics	Absolute number of patients *n* = 102	*%*
Median age in years	33	(range: 18–57)
Histology		
Seminoma	13	13%
Non-Seminoma	89	87%
UICC stage at primary diagnosis		
UICC I	5	5%
UICC II	27	26%
UICC Stage III	70	69%
IGCCCG classification at primary diagnosis of disseminated disease		
Good	9	9%
Intermediate	26	25%
Poor	67	66%
Localization of the primary tumor		
Gonadal	74	73%
Extragonadal retroperitoneal	16	16%
Extragonadal mediastinal	11	10%
Cerebral	1	1%
Time of stem cell mobilization		
First-line treatment	25	24%
Second-line treatment	58	57%
Third or consecutive line treatment	19	19%
Mobilization regimens		
G-CSF once daily s.c.	52	51%
G-CSF twice daily s.c.	39	38%
PegG-CSF	11	11%
Any of the above plus plerixafor	18	18%

### Induction chemotherapy and stem cell mobilization

SCM was administered as a part of first, second or consecutive treatment lines in 25 (25%), 58 (57%) and 19 (19%) patients, respectively. All patients received one cycle of mobilization chemotherapy with cisplatin, ifosfamide and etoposide (VIP) (*n* = 40) or paclitaxel, ifosfamide and cisplatin (TIP) (*n* = 14) or paclitaxel and ifosfamide (TI) (*n* = 48). Patients were mobilized with either subcutaneous injections of G-CSF (Neupogen®) 48 million IE once daily (group 1) (*n* = 52), G-CSF twice-daily (group = 2) (*n* = 39), or PegG-CSF (Neulasta®) 6 mg (group 3) (*n* = 11). The decision concerning different HSCs stimulation approaches was up to the preference of the treating physician. G-CSF was commenced after a median of two days (range: 1–3) post mobilization chemotherapy and PegG-GCF was administered throughout day one after completion of mobilization chemotherapy. SCM was conducted after a median of eight days (range: 5–11) after initiation of stem cell stimulation. The average number of G-CSF doses was 10.3 injections (range: 6–23) in group 1, 13.5 in group 2 (range: 6–28) and one in group 3. To achieve successful SCM 18 patients (6%) received additional plerixafor (Mozobil®) 20 mg fixed dose due to an insufficient CD34+ count prior or during the scheduled apheresis day (Table 2).

**Table 2:** Basic characteristics concerning different strategies of stem cell apheresis.

Mobilization method	Patients	Median number of filgrastim injections	Median days of apheresis (range)	Median number of CD34*cells	Patients with successful stem cell mobilization (%)
G-CSF once daily	52	10	2.15 (range: 1–4)	11.39 * 10^6^ (range: 2.2–38.6)	77
G-CSF twice daily	39	14	2.2 (range: 1–4)	13.2 * 10^6^ (range: 1.0–70.0)	69
PegG-CSF	11	1	2.54 (range: 1–4)	11.4 * 10^6^ (range: 5.6–18.8)	90

### Outcome of different approaches of stem cell mobilization

Of 102 patients, 77 patients (75%) achieved a successful amount of CD34+ cells. Patients underwent a median of two days of apheresis for stem cell collection (range: 1–4 days). The median amount of collected CD34+ cells per kg body weight per patient was 12.06*10^6^/kg (range: 1.0–70.0*10^6^ CD34+ cells/kg). Group 1 achieved a median of 11.39*10^6^ CD34+ cells/kg (range: 2.2–38.6*10^6^ cells/kg of body weight), group 2 a median of 13.2*10^6^ CD34+ cells/kg (range: 1.0–70.0*10^6^ cells/kg) and group 3 a median of 11.4*10^6^ CD34+ cells/kg (range: 5.6–18.8*10^6^ cells/kg). A once-daily injection of G-CSF resulted in a median apheresis duration of 2.15 days, a twice-daily injection of 2.2 days and a single PegG-CSF injection of 2.54 days, respectively. Concerning the SCM outcome of group 1, group 2 and group 3 successful SCM was achieved in 40 of 52 patients (77%) in group 1 versus 27 of 39 patients (69%) in group 2 versus ten of eleven patients (91%) in group 3, respectively (*p* = 0.344). Mobilization chemotherapy and SCM was conducted as a part of the first-line chemotherapy in 25 (25%), as second line in 58 (57%) and as consecutive chemotherapy treatment lines in 19 patients (18%), respectively. SCM was successful in 24 of 25 patients (96%) when conducted during first-line treatment versus 41 of 58 patients (71%) receiving SCM during second line and 12 of 19 patients’ (63%) when conducted during consecutive treatment lines (*p* = 0.016). Concerning the mobilization chemotherapy regimens 36 of 55 patients (65%) achieved successful SCM under TI versus three of seven (42%) under TIP versus 37 of 40 (93%) under VIP (*p* = 0.02). Of note, all patients who received SCM as first-line chemotherapy received VIP as mobilization chemotherapy (Table 3).

**Table 3:** Association between patient characteristics and sufficient stem cell harvest (≥8 * 10^6^ CD34+ cells/kg).

Factor	Successful apheresis	*P value*
Above versus below median age	78% vs. 77%	0.807
UICC I vs. II vs. III at initial diagnosis	80% vs. 74% vs. 74%	0.951
Seminoma vs. Non-Seminoma	77% vs. 74%	0.831
Treatment lines prior apheresis 0 vs. 1 vs. >2	100% vs. 69% vs. 63%	0.016
Mobilization treatment TI vs. TIP vs. VIP	65% vs. 42% vs. 93%	0.02
Mobilization with 1x/d G-CSF vs. 2x/d G-CSF vs. PegG-CSF	75% vs. 69% vs. 90%	0.357

### Use of plerixafor

Additional plerixafor was administered in 18 patients (18%) due to an insufficient count of HSCs during SCM. Here eight of 18 patients (44%) achieved successful SCM after using plerifaxor (Table 2). In group 1, eleven patients (21%) received 13, in group 2 six patients (15%) received seven and in group 3 one patient (9%) received one injection of plerixafor, respectively. In group 1, six of 11 patients (55%), in group 2 one of six patients (17%) and in group 3 one of one patient (100%) was able to achieve sufficient SCH with an additional administration of plerixafor.

### Survival analysis

**Fig. 1:**
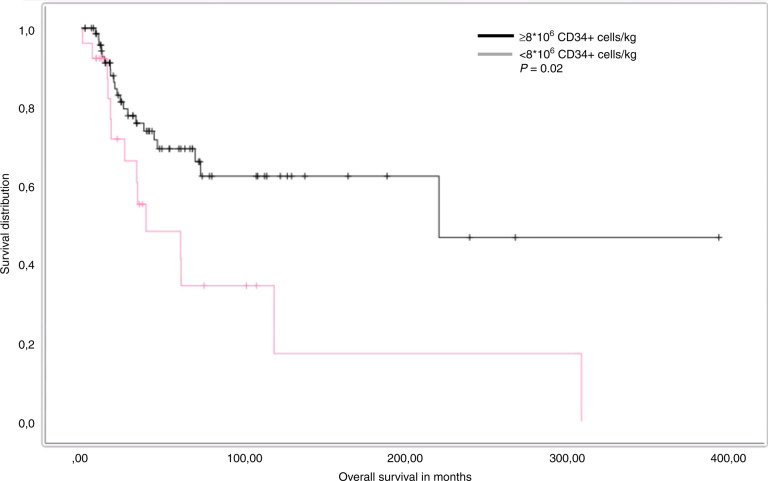
Patients achieving successful stem cell mobilization considered as ≥8 * 10^6^ CD34+ cells/kg demonstrated an improved outcome concerning OS.

The five-year OS rate from the timepoint of first diagnosis was 60%. The following variables were evaluated as potential prognostic markers: age (above vs. below median), IGCCCG (good vs. intermediate vs. poor), localization of the primary tumor (extragonadal vs. gonadal), histology (seminoma vs. non-seminoma) and stem cell apheresis outcome (≥8*10^6^ CD34+ cells/kg vs. <8*10^6^ CD34+ cells/kg). In univariate analysis successful SCM was the only significant prognostic factor, associated with a 5-year OS rate of 68% versus 45% favoring patients with successful SCM (*p* = 0.02). Further details concerning survival analysis are described in Table 4 and Fig. 1.

**Table 4:** Results of univariate analysis analyses of OS.

Factor	Difference 5-year OS rate	log- rank
		*p* value
Localization: gonadal versus extragonadal	69% vs. 52%	0.170
IGCCCG good vs. intermedia vs. poor	86% vs. 53% vs. 50%	0.580
Primary seminoma vs. non-seminoma	58% vs. 50%	0.787
Age above vs. below median	63% vs. 66%	0.679
Apheresis ≥8 * 10^6^ CD34+ cells/kg vs. <8 * 10^6^ CD34+ cells/kg	25% vs. 75%	0.012

### Cost-effectiveness analysis

The estimated costs were 189,30 € for one dose of G-CSF, 1.650,66 € for one dose of PegG-CSF and 7.309 € for one dose of plerixafor. In group 1 the median amount of daily G-CSF injections was 10 until successful SCM and apheresis was conducted, in group 2 14 and in group 3 one injection of 6 mg PegG-CSF. Further injections of plerixafor were conducted 13, 7 and one times in group 1, 2 and 3, respectively. The total amount for mobilization treatment for each group were estimated to be 193.453 € for group 1, 154.513 € for group 2, and 25.459 € for group 3, respectively. Considering the number of patients, the total costs per patient for mobilization treatment were 3.720 € for group 1, 3.961 € for group 2, and 2.314 € for group 3, respectively.

## Discussion

High-dose chemotherapy has been adopted as the standard of care in numerous GCC expert centers across the world, given the higher efficacy compared to conventional salvage chemotherapy in relapsed patients suggested by retrospective data. Moreover, as outlined in the recently published German Clinical Practice Guideline [15] nonseminomatous GCC patients with an extremely poor prognosis (i.e., mediastinal primary, insufficient marker decline on first-line chemotherapy, and/or the presence of brain metastases) may benefit from first-line HD-Cx [16–18]. Therefore, successful SCM and apheresis is mandatory to conduct subsequent HD-Cx with autologous stem cell support, but data on SCM approaches in GCC patients are scarce.

In our cohort 75% of the patients achieved successful SCM with consecutive apheresis. The only variable which was significantly associated with SCM success was the treatment line during which SCM was performed. Here, SCM outcomes were more successful if conducted during first-line treatment as compared to SCM outcomes during second or consecutive treatment lines. This may be explained due to the lower exposure of prior myelotoxic agents [19, 20].

Overall, in our patient population three different regimens of SCM were detected. For SCM patients received either G-CSF once daily, twice daily or a single dose of PegG-CSF. Our results demonstrated that the type of mobilization regimen did not significantly impact the SCM outcome, but as group 2 received G-CSF twice daily, an inferior impact concerning costs and effort was visible. Here previous analyses did also not find a benefit concerning a twice daily injection of G-CSF when SCM was attempted [21, 22]. Moreover, in our analysis a single injection of PegG-CSF was as effective as compared to multiple G-CSF administrations. Concerning costs and effort, a single injection of PegG-CSF is probably a more patient friendly approach and according to our calculation associated with lower health care costs. Even though our PegG-CSF cohort was small, most of the existing data which compare the cost effectiveness of PegG-CSF versus G-CSF provide similar results revealing a financial benefit favoring PegG-CSF with at least similar outcomes concerning the prevention of neutropenic fever [23–26], or SCM and apheresis [27].

Concerning the survival analysis an adequate SCM was the only prognostic factor associated with a beneficial OS. This is comprehensible as in case of insufficient SCM the intended treatment regimens which reveal the highest chances to achieve cure cannot be conducted.

The limitations of our study were obviously the retrospective design, and in particular the unbalanced groups with different regimens concerning SCM. Moreover, the clinical variables tested in univariate analysis such as localization of the primary, IGCCCG stage, and histology could be dependent on each other.

In conclusion, we were able to provide new insights concerning different SCM approaches in a large cohort of GCC patients. Overall, most of the patients achieved a successful SCM even during later treatment lines, irrespective of the type of induction chemotherapy or SCM regimens. However, the success rate of apheresis decreases with the amount of prior chemotherapy treatment lines. Concerning the different SCM regimens investigated, the use of G-CSF twice daily was associated with higher expenses, but without any benefit concerning efficacy, while single dose PegG-CSF was the most inexpensive approach associated with a SCM success rate of 90%. In case of an insufficient CD34+ count during mobilization treatment, plerixafor enabled a successful SCM in 44% of the patients.
